# Extracellular Vesicles: A Novel Tool in Nanomedicine and Cancer Treatment

**DOI:** 10.3390/cancers14184450

**Published:** 2022-09-14

**Authors:** Aikaterini Stavrou, Angelica Ortiz

**Affiliations:** Department of Medicine, Division of Environmental Medicine, New York University Grossman School of Medicine, New York, NY 10010, USA

**Keywords:** extracellular vesicles, cancer, cancer treatments, exosomes, drug delivery, protein delivery, nanoparticles, gene delivery, cancer vaccine, mesenchymal stem-cell-derived extracellular vesicles

## Abstract

**Simple Summary:**

Extracellular vesicles (EVs) are plasma-membrane-encased particles with various biomolecules. Recent studies have demonstrated that EVs play a role in homeostasis and disease progression, and therefore may be important disease biomarkers. In cancer, EVs mediate inflammatory responses, oxidative stress, and contribute to altering the microenvironment. Additionally, EVs function as mediators in neurodegenerative diseases. Interestingly, EVs also promote stem cell differentiation, intercellular communication, and wound healing. These functions suggest that EVs can be utilized in medicine as therapeutic tools. Moreover, their endogenous nature and ability to carry intact biomolecules of different sizes to their target site due to their lipid bilayer makes them perfect drug transport systems that can be utilized in the treatment of many diseases, with higher efficacy and fewer side effects than other treatments as they can only target diseased cells and not healthy nearby cells, which occurs in conventional chemotherapy, for example. As such, their role in drug delivery has great potential.

**Abstract:**

Extracellular vesicles are membrane-bound vesicles released by cells to mediate intercellular communication and homeostasis. Various external stimuli as well as inherent abnormalities result in alterations in the extracellular vesicle milieu. Changes to cells result in alterations in the content of the extracellular vesicle biogenesis, which may affect proximal and distal cells encountering these altered extracellular vesicles. Therefore, the examination of changes in the extracellular vesicle signature can be used to follow disease progression, reveal possible targets to improve therapy, as well as to serve as mediators of therapy. Furthermore, recent studies have developed methods to alter the cargo of extracellular vesicles to restore normal function or deliver therapeutic agents. This review will examine how extracellular vesicles from cancer cells differ from normal cells, how these altered extracellular vesicles can contribute to cancer progression, and how extracellular vesicles can be used as a therapeutic agent to target cancer cells and cancer-associated stroma. Here we present extracellular vesicles as a novel tool in nanomedicine.

## 1. Introduction

Various studies have identified and characterized types and subtypes of extracellular vehicles (EVs). The main three are exosomes, microvesicles, and apoptotic bodies, which are mainly identified by size. The pathways and conditions by which each type is generated and secreted by cells varies. Moreover, studies have demonstrated various subtypes to further characterize each of the EV types. 

Exosomes are the smallest, with diameters ranging from 30–150 nm (Table 1) [1]. The generation and release of exosomes requires regulated pathways. Exosome biogenesis requires endosomal sorting complex (ESCRT) machinery and intraluminal vesicle (ILV) formation via the inward budding of the endosomal membrane during the process of multivesicular endosome (MVE) maturation [2]. The endosomes are composed of a membrane compartment that functions as a shorting system for the various ILVs, directing them to their intended target sites, which are either the lysosome for degradation or the cell surface membrane for either extracellular secretion via exocytosis or recycling [3]. During the early endosome phase of exosome biogenesis, fusion occurs between early endosomes and endocytic vesicles, resulting in the incorporation of their content into exosomes [3]. Late endosomes (also known as multivesicular bodies or MVBs) and recycling endosomes are then formed via a chain of transformation processes, where the internalized cargo is sorted into small vesicles based on their fate (degradation or excretion/recycling) ranging from 30 nm to 100 nm, also referred to as ILVs [3]. In the last stage of this process, depending on their fate, the late endosomes will either fuse with the lysosomal membrane for degradation or will fuse with the plasma membrane and be secreted into the extracellular space as exosomes [3]. This process also includes multiple proteins that function as mediators and are currently in use as exosome markers for selective isolation [3]. Tetraspanin proteins such as CD9 and CD63 are a good example, as they are involved in the formation of ILVs by reorganizing the lysosomal membrane into specialized regions. The ESCRT proteins play unique roles in the different stages of the process, describe in more detail by Akers et al., 2013 [3]. Alix and TSG101 are some other proteins strongly associated with exosome biogenesis that are used as exosome markers [3]. There are also ESCRT-independent mechanisms by which exosomes can be formed, that are thus not associated with Alix and TSG101 [3]. CD63 is the strongest exosome marker; however, it is also implicated in other processes [3]. As such, it is vital to identify a more reliable exosome marker for precise characterization. 

Microvesicles, are moderately sized EVs with sizes ranging from 50–2000 nm (Table 1). As some microvesicles can be of similar size to exosomes, further characterization is required to distinguish them based on their distinct mechanisms of biogenesis [3]. Unlike exosomes, microvesicles are nonapoptotic blebs that shed directly from the plasma membrane. They are also called ectosomes or microparticles [5]. Microvesicles are generated via a dynamic interplay involving the redistribution of phospholipids and the contraction of cytoskeletal proteins in a nonuniform and asymmetric way, resulting in the formation of microdomains, and thus, membrane budding and vesicle formation via outward shedding of the plasma membrane [3]. The changes in the lipid distribution are attributes to changes in the activity of flippase, translocase, and scramblase [5]. The cytoskeletal changes required to generate the blebs are regulated by ADP-ribosylation factor 6 (ARF6) and the activation of small GTPase Ras homolog gene family member A (RhoA) and Rho-associated coiled-coil-containing kinases [5]. A subtype of microvesicles are arrestin domain-containing protein 1-mediated microvesicles (ARMMs), which bud directly from the plasma membrane and contain active NOTCH receptors [6]. Similar to exosomes, ARMMS carry multiple ESCRT proteins and TSG101, but also carry arrestin domain-containing protein 1 (ARRDC1) and some carry NOTCH2 receptors [6].

Apoptotic bodies tend to be the largest of the EV types. Apoptotic bodies can range from 500 nm to as large as 5000 nm in diameter, and are mainly released by cells undergoing apoptosis (Table 1) [5,7,8]. Apoptotic bodies play an important role in the downstream process of apoptosis. They are released into the extracellular space via membrane protrusions/blebs commonly occurring in apoptotic cells; a more detailed mechanism of biogenesis has been described by Caruso and Poon, 2018 [8,9]. Exosomes and microvesicles have been extensively studied with regards to their role in disease and normal physiology, as well as their content and biogenesis pathways, with exosomes being the most extensively studied EVs. On the other hand, only recently has more interest been shown in the research of apoptotic bodies regarding their role in diseases. That is because they have a large molecular pool, and it has been found in numerous studies that apoptotic bodies, in contrast with other EV types, can carry a large amount of RNA, as described by Bayraktar et al., 2017 [10]; thus, they could have potentially devastating effects on recipient cells. Therefore, even though they were previously thought of as garbage disposal sacs, current research has shown that it is crucial to investigate their role in maintaining normal physiology as well as that of diseases such as cancer, as they could potentially play a role in anticancer immunity according to Battiselli and Falcieri, 2020 [8].

EVs can be found in various biological tissues and fluids, as all cell types release EVs, though in different number, size distribution, and content depending on state of the cell. Upon release, EVs play an important role in mediating cell–cell communication either locally or at distal sites; i.e., they elicit systemic effects. EVs carry a variety of lipids and proteins on their surface that add to their uniqueness; this may contribute to their function or be used as a marker to characterize their cell of origin [11]. For example, ovarian cancer cells are often enriched with glycoproteins on their plasma membrane, and thus release exosomes enriched with glycoproteins [11]. Once exosomes reach their target cell, they either trigger intracellular signaling by direct interaction with cell surface receptors via ligands presented on the EV’s surface, or they can be taken up by plasma membrane fusion or via caveolin/clathrin-mediated endocytosis (main route), phagocytosis, and pinocytosis, thus entering the cells [11]. One example of direct interaction involves exosomes derived from dendritic cells; these exosomes express ligands, such as TNF, that bind their receptors to tumor cells, thus activating caspase for apoptosis [11].

The content of exosomes, and EVs in general, is indicative of the cell of origin as well as the health of the cell of origin [11]. Lipid rafts that are present in exosome membranes can facilitate the membrane fusion internalization process [11]. Furthermore, it is suspected that the low pH in the tumor microenvironment, and thus the higher rigidity, and increased sphingomyelin could potentially facilitate exosome fusion [12]. Once exosomes are internalized, they release their contents. This process is described in more detail by Gurung et al., 2021 [11]. Once internalized, EVs can release their cargo into the receptor cell, after which they can reach the lysosome for degradation or be recycled and re-released back into the extracellular space [13]. For the recipient cell’s function to be affected by the EVs, either positively or negatively, their cargo must be released upon internalization and escape the endosomal pathway; one such example was described by Valadi et al., 2007 [14,15]. The processes of the recycling and re-release or EVs and their intact cargo was demonstrated via O’Brien et al., 2022 [15]. However, as mentioned earlier, EVs can also affect target cells functionally without being internalized, by acting on cell surface receptors. EVs have been identified as important mediators of cell–cell communication and in maintaining homeostasis, by delivering their specific cargo into recipient cells. One of these functions involves inflammation and cell death; however, these processes are not only part of normal pathophysiology, but also of diseases, making EVs important mediators of disease progression. There are many diseases in which EVs participate significantly in their progression. One example is that, in neurodegenerative diseases, EVs can carry distinct miRNAs that can be used as biomarkers for Parkinson’s [16] or misfolded disease-associated proteins [17]. Furthermore, EVs have been found to play an important role in autoimmune disorder pathogenesis [18], heart disease [19,20], and many other diseases. EVs play a vital role in cancer initiation, promotion, progression, and therapy resistance, and their role in each of these aspects has widely been studied in recent years.

## 2. The Role of Extracellular Vesicles in a Healthy Organism

**Figure 1:**
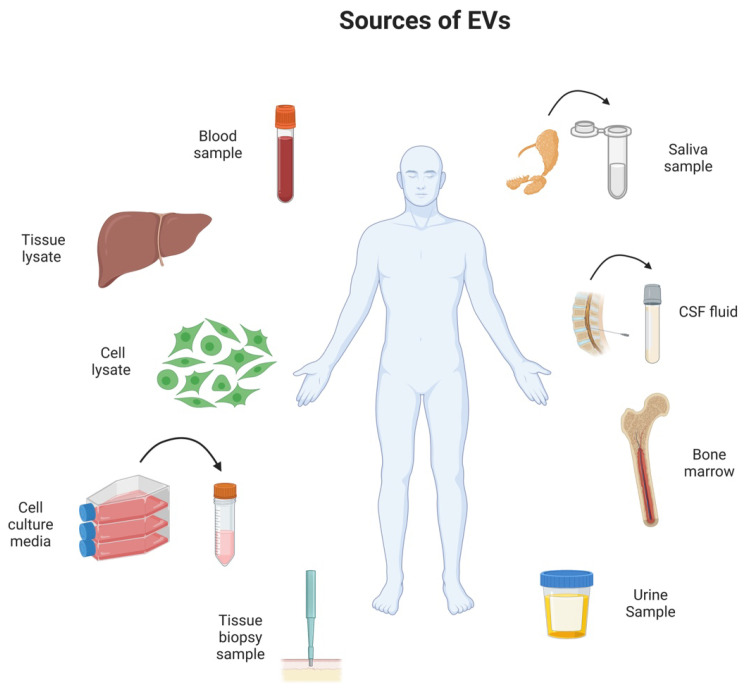
Schematic illustration depicting sources for EV collection. EVs can be isolated from various biological samples including bone marrow, cerebrospinal fluid (CSF), blood, urine, cell lysate, tissue lysate, cell culture media, and saliva and tissue biopsy.

EVs play a vital role in maintaining homeostasis as they are released by widespread biological processes and are found in many biological fluids, such as urine, blood, breast milk, bronchoalveolar lavage fluid, saliva, amniotic fluid (Figure 1) [21]. Exosomes are the EVs that are most intensively studied in terms of their roles in normal physiology and homeostasis. EVs are thought to play a role in many biological processes, such as immunity, protein clearance and signaling transduction [21]. It has been found that extracellular vesicles carry, among their cargo, a repertoire of biomolecules, such as RNAs, proteins, lipids, and more, some of which act as queues for the target site the EVs are destined for [22]. EV cellular communication can be monodirectional or bidirectional. During monodirectional communication, a cell creates and secretes EVs to the extracellular fluid. Those EVs have a target site they need to reach that is either cellular or tissue specific [22]. Once the target site is reached, their cargo is released, and elicits signaling events within the acceptor cell/tissue that can be highly specific as a response to the donor/EV-secreting cells, resulting in phenotypic changes, altered immune responses, and more, with the purpose of either maintaining cellular homeostasis or promoting disease [22,23]. However, upon incorporation, or in response to the EVs, the recipient cells may undergo changes in the biogenesis and secretion of EVs or other factors; this is an example of bidirectional communication. The feedback can be negative or positive; for example, it can suppress the secretion and/or assembly of donor EVs once the homeostasis is being restored (negative feedback loop). The monodirectional and bidirectional communication systems must be balanced in order to maintain a dynamic homeostatic state. However, there are several challenges remaining in fully understanding the mechanisms by which vesicle content is being packaged within the EVs. For example, it is well accepted that EVs are a heterogenous population and their content as well as size can significantly vary even though they have same origins. Another significant challenge is the lack of understanding the mechanisms by which EVs are able to be directed to their target site within the biological system, as well as the exact mechanisms by which their content is incorporated into the target cell [22]. It is essential for the research community to answer these questions and overcome these challenges as they are essential in the overall understating of the EV communication system in order to be effectively utilized for diagnostic and therapeutic purposes [22].

The role of EVs in maintaining cellular homeostasis is not only via content exchange between cells, but also function as a means for cells to excrete unwanted biomolecules, misfolded proteins, and toxic intracellular materials [23,24]. They have also been linked to regulating cell death during development via EVs from healthy cells regulating the cell death process in recipient cells [25]. Exosomes derived from stem cells are actively involved in tissue regeneration, cell differentiation maintain cellular homeostasis. For example, EVs from an injured tissue send the message via EVs to the mesenchymal stem cells in a bidirectional communication feedback loop where, in turn, mesenchymal stem cells release EVs loaded with the appropriate cargo in response to this, with the purpose of restoring the injured tissue [26]. Noncoding RNAs in EVs have also been shown to regulate the differentiation process of mesenchymal stem cells; more details on that topic are discussed in the review of Yan and Yu, 2022 [27]. 

As mentioned earlier in this section, the mechanisms by which EVs release their cargo into recipient cells are still not well understood. In an effort to shed light on this topic, the study of Joshi et al., 2020, [28] identified an intracellular site where EV content is released within the recipient cell, the endosomes/lysosomes. The study of Van den Broek et al., 2020, [29] showed that EVs derived from microglia have an influence on many biological pathways involved in cell survival and autophagy activation, in order to maintain cell homeostasis. Another study was able to show a novel molecular mechanism by which during heat shock, exosomes carrying molecular chaperones hsp40/hsp70 (which are physiologically secreted from cells) improved homeostasis of proteins, by improving the protein folding environment, thus restoring balance [30]. Finally, during pathogen infection, exosomes play a critical role in host defense against the pathogen. A good example was demonstrated in the study of Maemura et al., 2018 [31], where they examined lung derived exosomal microRNA content during infection with influenza virus, and found high mir-483-3p, which increased the concentrations of type I interferons and proinflammatory cytokines, indicating the vital role of EVs and their content in antiviral and inflammatory responses during infection with influenza virus, in an effort to restore healthy state and cellular homeostasis.

## 3. The Role of EVs in Disease State

Extracellular vesicles are not only important cellular communication mechanisms aiming to maintain homeostasis in healthy organisms, they also play an important role in disease progression. EVs have been implicated in many diseases, including neurodegenerative [32], autoimmune diseases [18], and more. However, for the purpose of this review, we will mostly focus on the role of EVs in inflammation and cancer. Inflammation plays an important role in both normal physiology and disease. EVs excreted from tumor cells have been found to promote cell survival and prevent cell death in recipient cells, where they release their cargo. One such example is shown in the study of Fonseka et al., 2019 [33]; these authors discovered that exosomes released from neuroblastoma cells overexpressing N-Myc promoted cell survival and chemoresistance in recipient cells that did not possess amplified N-Myc, and thus prevented cell apoptosis induced by doxorubicin and promoted cancer progression. EVs are also important mediators of inflammation via immune system modulation, as they contain a vast variety of chemokines, cytokines (e.g., CXCL2, TNF, and IL-1β), and other inflammation-related proteins [25,34].

**Figure 2:**
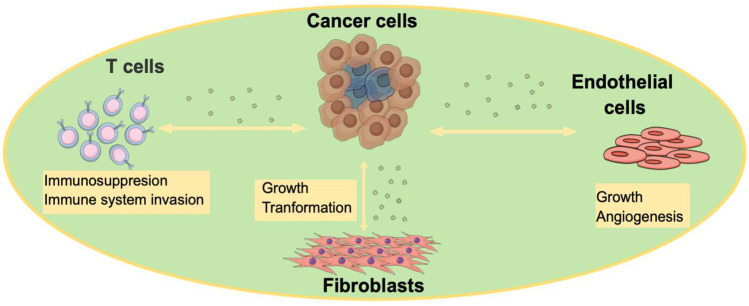
Schematic illustration depicting how cancer-cell-derived EVs affect various noncancer cell populations in the TME to favor cancer progression. Cancer cells secrete EVs to affect noncancer cells that comprise the TME (shaded area). Cancer-cell-derived EVs have the ability to increase the cell growth of both endothelial cells and fibroblasts within the TME. They also induce angiogenesis by targeting the endothelial cell machinery, and they transform fibroblasts into cancer-associated fibroblasts. Cancer-cell-derived EVs also target T cells to induce immunosuppression and promote immune invasion [1].

Oxidative stress is critical during cancer development, and EVs are highly involved in this process [35]. Moreover, EVs have the ability to promote cancer progression by altering the tumor microenvironment (TME) (Figure 2) to favor tumor growth and survival, as well as priming distance sites, (i.e., metastatic niche) prior to the arrival of metastatic tumor cells [35,36]. Tumor-cell-secreted EVs promote many aspects of cancer hallmarks, such as angiogenesis, epithelial-to-mesenchymal transition (EMT), immune invasion and/or escape, cell proliferation, matrix remodeling, and migration [35,37,38]. For example, in breast cancer, the study of Yang et al., 2011 [39] showed that microvesicles that are derived from macrophages promote invasion via the delivery of oncogenic miRNAs to breast cancer cells. Patients with cancer have been shown to have higher levels of circulating EVs (twofold in blood circulation) when compared to healthy individuals, as described by Kalluri, at al., 2016 [40]; however, the exact reason for this is not yet clearly understood. One suggestion is that ESCRT genes and other proteins and lipids that are involved in the biogenesis of EVs are highly elevated tumors [41]. Moreover, the extent to which EVs are elevated in cancer depends on the amplified genes in that cancer; for example, MYC and AURKB promote the release of some of the highest EV numbers [41]. It is also important to note here that each of these oncogenes alter the EV composition uniquely, especially the protein content, resulting in different EV cargo compositions not just between different types of tumors, but also within the same tumor [41]. 

Cancer-cell-derived EVs carry distinct pro-tumorigenic content that regulates several aspects of cancer promotion and progression. For example, cancer-cell-derived EVs carry pro-tumorigenic proteins such as growth factors (mutant EGFR) and oncogenes (e.g., KRAS) [42]. They also carry RNAs in order to target the recipient cell’s transcription machinery and promoter tumorigenesis [42,43]. Cancer-cell-derived EVs suppress immunity and immune surveillance against tumors by, for example, delivering TGFβ to recipient cytotoxic T cells, affecting their function [42]. Furthermore, they can also deliver FasL to activated T cells and induce their apoptosis, thus inhibiting their activation. Cancer-cell-derived EVs can also carry PD-L1 to other cancer cells that are PD-L1-deficient, thus inhibiting cancer immunity mediated by T cells and promoting immune invasion by the tumor cells [42]. One good example of this has been shown in relation to breast cancer in the research of Yang et al., 2018 [44]. Furthermore, it was found that, in glioblastomas, cancer cells undergoing apoptosis release apoptotic bodies carrying proteins such as RBM11 and small nuclear RNAs (snRNAs) to the recipient cells, resulting in more aggressive tumor phonotypes and drug therapy resistance by changing the splicing of the MDM4 and cyclin D1 mRNA in those recipient cells [43,45]. In colon cancer, exosomes have been found to transfer mutant oncogenes such as KRAS and SRC to other colon cancer cells lacking these mutations to further promote tumor invasion [43]. Tumor promotion is also facilitated partially by EVs in glioma cells, as they tend to transfer the oncogenic receptor EGFRvIII to other glioma cells lacking this receptor, thus, MAPK and AKT pathways become activated and promote anchorage-independent growth [43,46].

Tumor-cell-derived EVs presenting tetraspanins, such as TSPAN8, can also promote angiogenesis by regulating endothelial cell function, increasing their cell proliferation, and upregulating angiogenesis-associated gene expression during conditions of hypoxia [43]. Furthermore, ovarian-cancer-derived EVs are enriched in soluble E-cadherin/VE-cadherin heterodimers that have the potential to activate β-catenin signaling and the NK-kB pathway to induce angiogenesis in recipient endothelial cells [43,47]. In lung cancer, under hypoxic conditions, there is an increased number of exosomes released that are highly enriched in miR-23a, and, upon its delivery to endothelial cells, HIF-1α becomes overexpressed due to the inhibition of PHDs by miR-23a, resulting in increased angiogenesis and cancer progression [43,48]. EVs derived from tumor cells can also alter stromal cells to favor and promote tumor growth. In breast cancer, exosomes derived from the surrounding activated stromal cells contribute to tumor progression by delivering RN7SL1, thus activating RIG-1 signaling, resulting in an increase in inflammation [43]. Finally, cancer-associated fibroblasts (CAFs) release exosomes highly enriched in ADAM10, thus increasing GTPase RHOA and enhancing cancer cell motility. Moreover, ADAM10, once delivered to recipient cancer cells via CAF-derived exosomes, can maintain cancer cell stemness in several tumors via Notch signaling pathway activation [43]. The ability of tumor-cell-derived EVs to transform normal cells is well described in the review of Chulpanova et al., 2002 [49], highlighting recent advances in the field. EVs from mesenchymal stem cells (MSCs) have critical roles within the TME that can be anti- or pro-tumorigenic. More detail on the roles of MSC-derived EVs in cancer can be found in the review of Gilazieva et al., 2022 [50]. Overall, we observe that EVs play a critical role in disease progression, and better understanding of this role in both disease and healthy environments, filling the current gaps in literature, is vital in helping us to better utilize them in medicine for diagnostic and treatment purposes.

## 4. EVs in Cancer Therapy

Given the part EVs play in disease and in maintaining homeostasis, they have a promising role in cancer therapy, with hopes of limiting the toxicity of current anticancer drugs and increasing their efficacy. As mentioned in the previous section of this review, cancer cells release a large amount of EVs compared to normal/healthy cells, and these EVs have distinct cargos based on the cancer type, as well as the particular cancer cell releasing them (e.g., chemotherapy-resistant vs. chemotherapy-sensitive cells). We also know that EVs are able to carry biomolecules to other cells. These biomolecules are well preserved and stable within the EVs, as they are protected by the lipid bilayer membrane of EVs from enzymatic degradation. As such, EVs can be targeted in cancer treatment, for example, by potentially inhibiting the release of extracellular vesicles derived from tumor cells; especially tumor cells that are therapy resistant. Using a GW4869 inhibitor could improve treatment outcomes, as therapy-resistant cells would no longer be able to influence surrounding therapy-sensitive cancer cells [51,52]. Furthermore, when EV release from cancer cells is inhibited, they will not be able to influence surrounding normal cells and transform them into cancer cells; thus, invasion into the surrounding healthy cells is blocked [53]. EVs from cancer cells can also interfere with cancer treatment by interacting directly with the chemotherapy drug and decreasing its efficacy. For example, in the study of Ciravolo, et al., 2011 [54], it was shown that EVs, in particular exosomes, released from HER2-positive breast cancer cells can bind to Trastuzumab and interfere with its antiproliferative effects on breast cancer cells, thus diminishing its efficacy. Given the above, inhibiting EV release from cancer cells could significantly increase the efficacy of cancer therapies. This can also be a therapy of its own, as by inhibiting these tumor-cell-derived EVs, you may also be inhibiting/suppressing tumor progression.

### 4.1. EVs as a Drug Delivery System

EVs are an excellent drug delivery system as they are naturally occurring messengers that deliver cargo to cells. Thus, they have an innate biocompatibility and are able to deliver a variety of cargo, and their cell surface can be modified to target a specific cell population. By targeting specific cell populations, EVs have the ability to limit the cytotoxic effects of chemotherapy drugs on healthy cells (i.e., the nonspecific cytotoxicity of chemotherapy drugs), limiting adverse effects while improving drug delivery and efficiency [55]. Moreover, EVs can be loaded with both hydrophobic and hydrophilic drugs, although the drug loading process into the EVs can vary [56]. Due to their composition (i.e., protein-containing lipid bilayer membrane), EVs are far more stable than liposomes and synthetic-polymer-based nanoparticles [56]. In some cases, exosomes carry CD47 to protect them against phagocytosis, resulting in more exosomes remaining in circulation compared to engineered liposomes [57]. Due to the stability of the exosomes, the exosomes carrying the therapeutic short interfering RNA targeting oncogenic KRAS-expressing cancer cells are more efficient that liposomes with the same RNA cargo [57]. One very important aspect of EVs that could solve a major problem in brain cancer treatments, is that they are able to pass through many physiological barriers within the body, including the blood–brain barrier (BBB) [58]. The BBB has posed a huge challenge in treating brain cancers, such as glioblastomas, as drugs cannot pass through and enter the brain. EVs derived from tissue-specific cells, in this case, from brain endothelial cells, have the ability to cross the BBB due to their natural surface protein composition [56]. For example, in the study of Yang et al., 2015 [56], they demonstrated that EVs derived from brain endothelial cells with cell surface tetraspanin CD63, when loaded with chemotherapy drugs such as paclitaxel and doxorubicin, can efficiently pass through the BBB and induce cytotoxic effects on brain cancer cells. Furthermore, in the research of Yang et al., 2015 [56], it was shown that both glioblastoma–astrocytoma cells (U-87 MG) and brain-endothelial-cell-derived EVs, exosomes in particular, loaded with doxorubicin and paclitaxel, significantly decreased cancer cell viability.

In the study of Hadla et al., 2016 [59], it was shown that exosomes loaded with Doxorubicin increased the drug’s therapeutic index while it decreased its cardiac toxicity. The exosomes were bioengineered to only target breast or ovarian cancer cells, while avoiding crossing with myocardial endothelial cells. In doing so, they were able increase the dose of doxorubicin for better efficacy of the drug against ovarian or breast cancer, while having no cardiotoxic side effects. This is a good example showing how EVs can increase drug specificity and efficacy, while decreasing cytotoxicity and side effects. 

### 4.2. The Role of EVs in Immunotherapy

**Figure 3:**
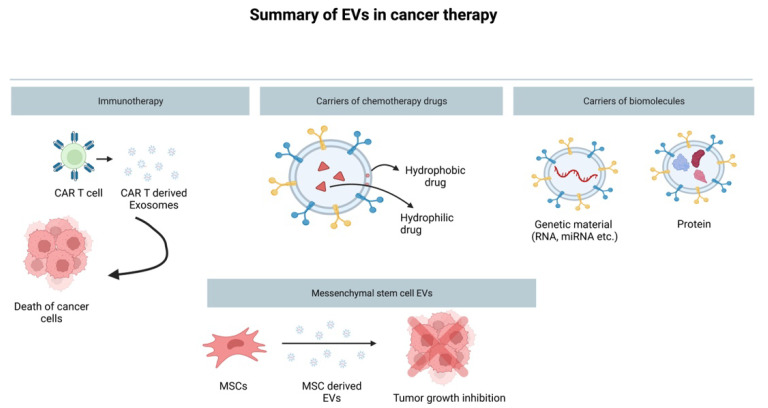
Schematic illustration summarizing the use of EVs in medicine as drug delivery systems. EVs can be used in immunotherapy, such as exosomes derived from CAR-T cells, can be effective at promoting cell death via immune defense activation [63]. EVs can also be loaded with chemotherapy drugs (both hydrophobic and hydrophilic), and can safely deliver the drug to the cancer cells intact due their lipid bilayer in which the drug is enclosed. Moreover, EVs can be loaded with biomolecules such as antitumorigenic miRNAs, long noncoding RNAs, mRNAs, and proteins (e.g., tumor suppressors). Finally, EVs derived from mesenchymal stem cells can be utilized as an anticancer treatment themselves due to their ability to suppress tumor growth.

Immunotherapy is a major player in cancer treatment, often given in combination with chemotherapy and radiation (conventional treatments) for optimal results. There is a lot of research demonstrating the critical role EVs can play in immunotherapy. Tumor-cell-derived EVs contain proteins that possess immunoregulatory functions, including Hsp70 and MHC-1 (major histocompatibility complex class I), and can interfere with T cell function [30]. Moreover, immune-cell-derived EVs, such as dendritic cells, could be a potential treatment method in cancer immunotherapy, since they can induce potent antitumor effects, and are thought to be a potential cell-free vaccine for tumor therapy [60]. The study of Andre et al., 2004 [61], demonstrated that exosomes derived from melanoma patients, in particular, exosomes from malignant effusions, have the potential to deliver melanoma antigens and tumor antigens to dendritic cells, and efficiently activate dendritic-cell-mediated cytotoxic T cell lymphocytes. The review of Pitt et al., 2016 [62], discusses phase I and phase II clinical trials with dendritic-cell-derived EVs for NSCLC treatment by mediating T cell and NK cell immune responses. Chimeric antigen-receptor-modified T cells, otherwise referred to as CAR-T, are a promising new type of immunotherapy in cancer because they can improve anticancer immune response; however, they have been associated with cytokine release syndrome, which poses a large obstacle in their potential use in immunotherapy [63,64]. However, CAR-T cells can release exosomes carrying the chimeric antigen receptor (CAR) that can affect cancer cells, thus stimulating dendritic cell activity and promoting antitumor immune response without the risk of causing cytokine release syndrome, making them the “ultimate attackers” against cancer (Figure 3) [63,64]. Finally, in the study of Morishita et al., 2016 [65], they proposed a tumor antigen adjuvant co-delivery system that is exosome based, formed by genetically engineering tumor-cell-derived exosomes with endogenous tumor antigens and immunostimulatory CpG DNA in their cargo as an effective immunotherapy treatment, eliciting specific antitumor immune responses. The review of Giacobino et al., 2021 [66], summarizes some of the current clinical trials of EV-based vaccines in the field of immunotherapy in cancer [66].

### 4.3. Mesenchymal Stem-Cell-Derived EVs and Their Cancer Therapy Potential 

Another emerging potential of EVs in antitumor therapy is the utilization of mesenchymal stem cell (MSC)-derived EVs. MSCs are part of the TME, and can contribute to tumor progression. MSCs are also involve in maintaining stem cell homeostasis, tissue repair, and differentiation. MSCs normally reside in the perivascular niches in all types of tissues, with the purpose of activating tissue-specific functions once activated during certain conditions [67]. Upon the recruitment of MSCs to damaged sites, they release exosomes containing immunomodulatory cargo, promoting angiogenesis and tissue repair by interacting with a vast range of cell types, including endothelial cells, immune cells, fibroblasts, pericytes, and more [67]. In invasive tumors where there is a lot of tissue injury due to the aggressive nature of the tumors, MSCs are recruited for tissue repair. As mentioned above, MSCs communicate with various cell types, and, in tumors, once they arrive at the TME, they also interact with cancer cells via the exchange of bioactive molecules through EVs, thus altering both of their functionalities [67]. Tumor-cell-derived exosomes can, in fact, turn MSCs into cancer-associated fibroblasts (CAFs) that would then further contribute to tumor progression [67]. It has also been shown that EVs released from MSCs to TME cells, can also contribute to tumor progression by aiding in angiogenesis, tumor growth and metastasis via the delivery of certain miRNAs in a paracrine manner [67]. They can also move cancer cells in a state of quiescence (dormancy). However, exosomes from MSCs can also have the opposite effect on cancer cells, leading to the inhibition of tumor growth and prevention of metastasis by activating antitumorigenic signaling pathways [67]. This contradictory role of MSC-derived EVs in tumors can be attributed to the heterogeneity of the MSC populations. MSCs are very sensitive to their environment, and their behavior as well as EV cargo and secretory activity can be altered due to physiochemical factors within their microenvironment (e.g., pH, oxygen availability, ion gradient), thus leading to their controversial functionality in cancer [67]. It is important to note here that within the same tumor, but at different sites, MSC-derived EVs can have opposite effects. Unfortunately, to date, the molecular signaling involved in the regulation of the MSC-derived-EVs’ effects on tumor development is not well understood, which poses difficulties in their utilization in cancer therapy. 

Even though the use of MSC-derived EVs as a cancer therapy alone is still not feasible due to the controversy around them, they are indeed a great drug delivery system in cancer therapy, as they have the ability to quickly migrate to tumor sites, much like MSCs themselves [68]. In clinical application, MSC-derived EVs as drug delivery systems are also great due to the MSCs’ high proliferation ability, and hence production of large EV amounts under suitable cell culture conditions. MSCs can be isolated from bone marrow, placenta, and the umbilical cord. As such, they have attracted a lot of interest as drug delivery systems. The study of You et al., 2022 [68], showed that EVs derived from MSCs were able to transfer lipocalin-type prostaglandin D2 synthase (L-PGDS) to the target/cancer cells, and thereby inhibit the growth of gastric cancer both in vivo and in vitro. Furthermore, the study of Sheykhhasan et al., 2021 [69], showed that miR-145 overexpressing adipose tissue MSC-derived exosomes have the potential to increase miR-145 levels in breast cancer cells, and thus inhibit metastasis and induce apoptosis by modulating several anticarcinogenic signaling pathways. The study of Sheykhhasan et al., 2021 [69], is an example of a system by which MSC-derived EVs can be modified via transfection. Paclitaxel, commonly used as a chemotherapy drug in cancer treatment, can be used to treat MSC. EVs can then be collected from paclitaxel-treated MSCs to be used as an effective treatment against breast cancer [70]. 

### 4.4. Methods of Loading EV Biochemical Molecules

As mentioned earlier in this review, both hydrophobic and hydrophilic molecules can be loaded within EVs due to the unique structure of the EV membrane, which has a hydrophobic space within the lipid bilayer and a hydrophilic surface. An example of some of the molecules that can be loaded are proteins, RNAs, DNA, and anticancer drugs. There are two main loading approaches when it comes to therapeutic EV cargos. One is exogenous, or direct, loading, where therapeutic cargos are loaded past isolation [71]. This type of loading is further subdivided into two categories: passive loading, where a therapeutic cargo is passively loaded into EVs via a co-incubation; and active loading, which requires EV-membrane disruption via electroporation or surfactant addition [71,72,73]. The other approach is endogenous loading, where a donor cell incorporates the therapeutic cargo into the EVs prior to their release, either via genetically engineering the donor cells or transient transfection of the donor cells [71]; for example, EV RNA loading following vector expression [74]. More details about the methods of loading EVs with biomolecules and pharmaceuticals can be found in Sutaria et al., 2017, Johnsen et al., 2014, and Busatto et al., 2021, [71,75,76].

Another important aspect of EV bioengineering for cancer therapy is altering the surface of EVs, also called EV surface functionalization [66]. In doing so, we can improve the targeting abilities of EVs, thereby improving their therapeutic applications. However, the required protocols are not as well established as they are with the loading of EVs. Thus, it is critical to develop appropriate protocols that will preserve the integrity as well as function of EVs, to better improve their potential medicinal use [66]. More details about the current methods of EV surface functionalization can be found in the review of Rayamajhi and Aryal, 2020, [77].

## 5. Conclusions and Future Directions

EV use in disease therapy has a lot of advantages, including the fact that EVs can be obtained from the patient themself, are noninvasive, have low toxicity and immunogenicity, have high stability in circulation, and can pass though many biological barriers [78]. EVs are also easy to isolate in large capacity, and are very stable due to their lipid bilayer membrane. There are several challenges that scientists and the medical community are facing with regards to the use of EVs as drug delivery systems. According to Johnsen et al., 2014 [75], it is essential that the proper donor cells are used to collect EVs, as well as the appropriate use of targeting peptides and drug-loading methods to achieve desired results. Furthermore, the route of administration of the engineered EVs plays a vital role in successful treatment, as every cancer is different [75]. Research has advanced greatly in this field; however, there are still many unknowns, and current designs need further refinement to in order for their use to be justifiable in a clinical setting. Much research has been conducted in the field of extracellular vesicles with regards to their cargo, heterogeneity, modes of biogenesis, and packaging. For example, some new advances in the field can be seen in the study of Maisano et al., 2022 [79], where they developed an new approach for identification, isolation, and characterization of disease-associated exosomes based on distinct antigenic reactivities that has a promising future in clinical use [79]. One of the major challenges to date is the lack of EV-type-specific markers, for example, markers that are only found on exosomes or only found on microvesicles, and so on. Another challenge, going even further, is identifying biomarkers associated with specific disease-derived exosomes. Such new advances in the field could be extremely beneficial, and could help establish the use of EVs in the medical field as biomarkers as well as drug delivery systems. As technologies advance and improvements are made in the isolation of various types and subtypes of EVs, our understanding of their roles in normal physiology and disease states will expand. Moreover, the manipulation of these membrane-bound vesicles can allow us to improve tissue regeneration, tissue function, or target diseased tissue.

## Figures and Tables

**Table 1:** Main EV categories.

Category	Size	Markers
Exosomes	30–150 nm [1]	CD63, CD9, ALIX, TSG101 [3,4]
Microvesicles	50–2000 nm [3]	ARF6, VCAMP3, Selectins, CD40 [3,4,5]
Apoptotic bodies	500–5000 nm [6,7]	TSP, C3b, Annexin V [3]
